# Sex‐Specific Determinants of Noncommunicable Diseases Among Respondents From Wealthier Households in Bangladesh

**DOI:** 10.1002/puh2.70461

**Published:** 2026-11-13

**Authors:** Sourav Roy, Raisha Binte Islam, Syed Toukir Ahmed Noor, Shuvongkar Sarkar, Md. Ismail Hossain

**Affiliations:** ^1^ Department of Statistics University of Dhaka Dhaka Bangladesh; ^2^ Department of Statistics Shahjalal University of Science and Technology Sylhet Bangladesh; ^3^ Statistics Program BRAC University Dhaka Bangladesh; ^4^ Department of Mathematics and Statistics Mississippi State University Mississippi State Mississippi USA

**Keywords:** body mass index (BMI), diabetes, hypertension, noncommunicable diseases, sex, wealth index

## Abstract

**Background:**

Noncommunicable diseases (NCDs), including hypertension and diabetes, have emerged as a major global public health concern in low‐ and middle‐income countries like Bangladesh. In light of this, the current study aims to identify the sociodemographic factors associated with NCDs among Bangladeshi men and women, with a particular focus on those from wealthier households, to gain a deeper understanding of NCD risk in a rapidly evolving health landscape.

**Methods:**

Data were used from the nationally representative Bangladesh Demographic and Health Survey (BDHS) 2022. A total of 2729 men and 3441 women from wealthier households were included in the analysis. NCD status was defined as the presence of hypertension, diabetes, or both, and the $\chi^{2}$ test of independence was used to assess associations between NCDs and background characteristics. Separate binary logistic regression models for both men and women were employed to estimate the effects of sociodemographic factors on NCDs.

**Results:**

This study estimated a higher prevalence rate of NCDs among women (41.3%) compared to men (37.4%). Both adult men and women had a higher association with NCDs, with the effect more noticeable among the age groups 30–44, 45–59, and 60+ years. Secondary and higher levels of education were less likely associated with NCDs among women, whereas men with higher levels of education had an increased association with NCDs. Overweight (aOR = 1.80) and obese (aOR = 4.08) men had a higher likelihood of association with NCDs with respect to normal body mass index (BMI), and a similar trend was found among overweight (aOR = 1.73) and obese (aOR = 3.00) women.

**Conclusion:**

This study shed light on the sex‐specific higher association with NCDs among rich Bangladeshi people. Increased age and higher BMI are associated with the development of NCDs among both men and women.

## Graphical abstract

This study identified that increased adult age, urban place of residence, and higher BMI are statistically significant associated factors of NCDs among wealthier households, evident from the BDHS 2022 data. The notable findings of this study are that men have a higher association with these factors compared to women.

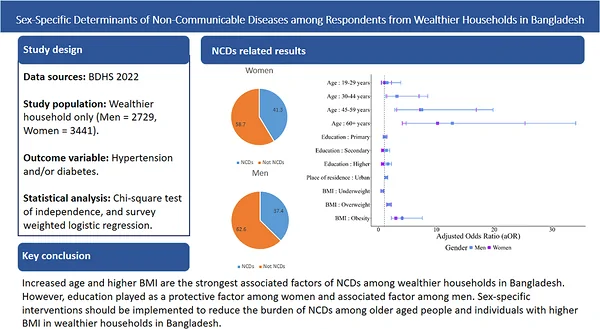

## 1 Introduction

Hypertension and diabetes are typically known as noncommunicable diseases (NCDs) because of their noninfectious nature [1, 2, 3]. NCDs are not like communicable diseases; they take a long time to develop and often occur silently. Once it is diagnosed as positive, it is not curable like infectious diseases, and the outcome is death. The disease needs to be controlled after diagnosis by taking medicine for the rest of one's life, which is an economic loss for the individuals [2, 3].

In recent years, the global burden of NCDs has been rising alarmingly. It was found in a study published by the World Health Organization (WHO) in 2024, at least 43 million people died due to NCDs across the world in 2021 [3], which is equivalent to 75% of the world's non‐pandemic‐related deaths [3]. Approximately three‐quarters (73%) of all NCD‐related deaths occurred in low‐ and middle‐income countries [3]. The study also found that 18 million people died due to NCDs before age 70 years in 2021, and 82% of these premature deaths occurred in the low‐ and middle‐income countries [3]. It was also found in their study that hypertension, diabetes, and overweight or obesity are key behavioral‐associated factors of NCDs [3]. Diabetes has reached pandemic levels, which is increasing by 70% each year since 2000. Nowadays, one out of nine adults across the world lives with diabetes, and this scenario will highly affect low‐ and middle‐income countries [4].

Bangladesh is a lower middle‐income country in the South Asian region and has been experiencing a burden of NCDs for the last few decades. As it was found in a study by WHO that 70% of all deaths in 2019 occurred due to NCDs, and one in five people at risk of dying due to NCDs are aged between 30 and 70 years [5]. Similarly, Ali et al. (2024) found in their study that there is an increasing trend of NCDs in Bangladesh. They found that the prevalence of diabetes increased by 1.8% between 2011 and 2017, whereas a noticeable increasing trend was observed in the prevalence of hypertension in the same period, which was 14% (26.4 in 2011 and 40.4% in 2017) [6]. It was also found in their study that wealthier individuals have 1.9 times greater prevalence ratio of diabetes relative to the poorest in 2011, whereas this ratio was found to be 2.6 times in 2017 [6]. Similarly, the study also reported that the richest people have a higher prevalence ratio (1.6 in 2011 and 1.2 in 2017) of hypertension compared to the poorest in the same time period [6]. The study also identified that the richest individuals are at 2.3 times higher likelihood for diabetes and 1.3 times increased chance for hypertension compared to the poorest people [6]. A recent study has identified that unhealthy food consumption, use of tobacco, and alcohol consumption are associated factors of NCDs, which increase blood pressure, high glucose level in blood, and high body weight, increasing the risk of the disease [7].

As a study found that wealthier individuals in Bangladesh have a higher risk of NCDs [6], a comprehensive study of the prevalence of NCDs among wealthier individuals in the country can provide a better understanding of the current epidemiology of the disease. Focusing on wealthier households is not a study convenience but a deliberate analytical choice grounded in epidemiological evidence. Multiple studies using Bangladesh DHS data have consistently demonstrated that individuals from the highest wealth quintiles face significantly greater odds of hypertension, diabetes, and their coexistence compared to lower wealth quintiles [6, 8, 9, 10], driven by sedentary lifestyles, overnutrition, and rapid economic transition. A study focusing on the lower educated population in Bangladesh similarly reported that wealthier families had a higher risk of NCDs, suggesting that the wealth–NCDs gradient persists across different subpopulation analyses [11]. This disproportionate yet comparatively understudied NCDs burden among wealthier households makes them a high‐priority subpopulation deserving targeted sex‐disaggregated investigation. This study aims to estimate the sex‐specific prevalence of NCDs in Bangladesh among the wealthier households and then quantify the effect of key sociodemographic characteristics that influence NCDs. The findings of this study will help policymakers to address the disease burden in the country among wealthier people.

## 2 Methods

### 2.1 Data Source and Sampling

The study was designed on data from the Bangladesh Demographic and Health Survey (BDHS) 2022, which ran from August to December 2022. A two‐stage stratified sample approach was utilized in BDHS 2022, with the Bangladesh Bureau of Statistics (BBS) constructing the sampling frame drawn from the 2011 Population Census. Using probability proportional to size, 675 enumeration areas (EAs), comprising 237 urban and 438 rural, were first selected. The second step, when 45 households per EA were chosen to provide reliable demographic and health data for both urban and rural populations, was based on the household listings inside these EAs. The final report of the BDHS 2022 offers more information on the sampling approach [12].

### 2.2 Sample Design

This study was extensively focused on the wealthier households in Bangladesh. The individuals classified under the richer and richest wealth index were included in this study, and the poorest, poorer, and middle wealth indices were excluded from the study. This restriction was intentional and theoretically grounded; prior evidence from Bangladesh has demonstrated that individuals in the richer and richest wealth quintiles carry a disproportionately higher burden of NCDs compared to lower wealth quintiles. The final dataset comprised 2729 (weighted) observations for men and 3441 (weighted) observations for women.

### 2.3 Dependent Variable

The dependent variable of this study focused on sex‐specific NCDs status among the wealthier households. To define the NCDs status, the American Heart Association guidelines were followed. An individual having either systolic blood pressure ≥140 or diastolic blood pressure ≥90, or taking medicine to control hypertension, was considered hypertension patient [13]. Similarly, if the fasting plasma glucose level of the respondents was ≥7.0 or if they were taking medicine for diabetes, they were treated as diabetic patients according to WHO guidelines [14]. Finally, if the individual has either hypertension or diabetes, or both, they are defined as NCDs Status 1 (present), otherwise 0 (absent).

### 2.4 Explanatory Variables

Several sociodemographic variables (as explanatory variables) were included in this study on the basis of the previous literature. Age, education, place of residence [11], region [11], marital status [15], and body mass index (BMI) of the individual were included in this study. Age group was categorized as young if the individual had less than 25 years, otherwise adult. The geographic region was categorized into four groups: Dhaka and Mymensingh as the central part, Rajshahi and Rangpur as the northern part, Chattagram and Sylhet as the eastern part, and Khulna and Barisal as the southern part [11]. Lastly, BMI was categorized on the basis of the classification provided in the BDHS 2022 report [12], which was similar to the Centers for Disease Control guidelines [16].

### 2.5 Statistical Analysis

A univariate analysis was conducted to describe the sociodemographic characteristics of the included individuals. Further, to examine the relationship of NCDs status with several sociodemographic factors, a chi‐square test of independence was applied. Finally, two distinct binary logistic regression models were fitted for men and women to estimate sociodemographic effects on NCDs in Bangladesh. A *p* value obtained less than 0.05 was considered statistically significant. All statistical analyses were performed in R version 4.5.1.

To account for the complex survey design of the BDHS 2022, all analyses incorporated sampling weights, clustering, and stratification using the “survey” package in R. The individual sample weights, primary sampling units, and stratification variables were applied to obtain nationally representative estimates and correct standard errors.

### 2.6 Ethical Consideration

This study used de‐identified secondary data from the DHS websites (https://www.dhsprogram.com/data/available‐datasets.cfm/). As the data were publicly available and did not contain any personally identifiable information, ethical approval was not needed. All participants provided informed consent, and the original surveys were carried out in accordance with ethical standards and approved by the appropriate institutional review boards.

## 3 Results

The sociodemographic characteristics of the study population are presented in Table 1. Around one‐third of the men (32.9%) and women (33.3%) were in age group of 30–44 years. A large portion of the men (secondary = 35.2% and higher = 33.1%) and women (secondary = 40.9% and higher = 22.1%) included in this study were educated. Similarly, more than half of the men (53.5%) and women (55.2%) were from urban areas, whereas a representative and approximately equivalent proportion of respondents were collected from the southern, northern, and eastern parts of the country. Particularly, the majority of the respondents were from the central parts of the country. However, the majority of the men (79.8%) and women (79.4%) included in this study were married. Finally, most of the people included in this study have normal (men = 58.9% and women = 45.6%) and overweight (men = 25.8% and women = 34.1%) BMI.

**TABLE 1:** Sociodemographic characteristics of the study individuals.

Variable	Categories	Women	Men
		*n* (%)	*n* (%)
Age (years)	≤18	109 (3.3)	81 (2.8)
	19–29	1048 (30.1)	629 (22.9)
	30–44	1118 (33.3)	893 (32.9)
	45–59	702 (20.2)	630 (23.0)
	60+	464 (13.1)	496 (18.4)
Education	Illiterate	594 (18.1)	316 (11.9)
	Primary	629 (18.9)	532 (20.8)
	Secondary	1379 (40.9)	976 (35.2)
	Higher	839 (22.1)	905 (32.1)
Residence	Rural	1616 (55.2)	1227 (53.5)
	Urban	1825 (44.8)	1502 (46.5)
Region	Central	903 (38.4)	706 (38.6)
	Southern	802 (17.3)	673 (18.9)
	Eastern	985 (24.7)	725 (21.2)
	Northern	751 (19.5)	625 (20.5)
Marital status	Unmarried	273 (6.5)	518 (18.0)
	Married	2676 (79.4)	2149 (79.8)
	Widowed/Divorced/Separated	492 (14.1)	62 (2.2)
BMI	Underweight	286 (8.0)	318 (12.0)
	Normal	1568 (45.6)	1608 (58.9)
	Overweight	1169 (34.1)	711 (25.8)
	Obesity	418 (12.3)	92 (3.3)

Abbreviation: BMI, body mass index.

Men showed a consistently higher prevalence of NCDs in the southern, central, and northern regions of the country compared to men (Figure 1). However, women had a lower prevalence of NCDs in the eastern region compared to men.

**FIGURE 1:**
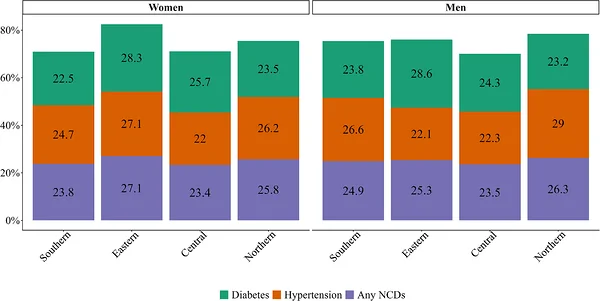
Prevalence of hypertension, diabetes, and NCDs across divisions by sex. NCDs, noncommunicable diseases.

A chi‐square test of independence was performed to identify the relevant sociodemographic factors responsible for developing NCDs between both men and women, which is presented in Table 2. The prevalence of NCDs was estimated 37.4% among men (1049 men out of 2729), whereas 41.3% among women (1420 women out of 3441) in Bangladesh. Age, education, place of residence, marital status, and BMI were found to be statistically significant (*p* value < 0.05) associations with NCDs status among both men and women. These demographic factors were further included in the binary logistic regression model to quantify the effect of each sociodemographic characteristic for developing NCDs. Women and men having age groups of 45–59 years (women = 63.6% and men = 51.3%) and 60+ years (women = 68.2% and men = 58.9%) had the highest prevalence of NCDs compared to their counterparts. Men (42.7%) and women (62.3%) with no formal education had the highest prevalence of NCDs compared to their counterparts. Women residing in rural areas (43.7%) had a higher prevalence of NCDs than women who lived in urban areas, whereas men had a higher prevalence of NCDs in urban areas (40.3%) than men residing in rural areas. The highest prevalence of NCDs was also observed among the widowed, divorced, or separated women (62.2%) and men (57.3%). As expected, obese women (61.4%) and men (65.7%) had the highest prevalence of NCDs compared to their counterparts.

**TABLE 2:** Prevalence and association of the sociodemographic characteristics with noncommunicable diseases (NCDs) status.

Variable	Categories	NCDs among women			NCDs among men		
		No, *n* (%)	Yes, *n* (%)	*p* value	No, *n* (%)	Yes, *n* (%)	*p* value
Overall		2021 (58.7)	1420 (41.3)		1680 (62.6)	1049 (37.4)	
Age (years)				<0.001			<0.001
	≤18	98 (87.1)	11 (12.9)		73 (90.7)	8 (9.3)	
	19–29	876 (83.7)	172 (16.3)		520 (83.7)	109 (16.3)	
	30–44	647 (57.5)	471 (42.5)		586 (67.1)	307 (32.9)	
	45–59	261 (36.4)	441 (63.6)		297 (48.7)	333 (51.3)	
	60+	139 (31.8)	325 (68.2)		204 (41.1)	292 (58.9)	
Education				<0.001			0.077
	Illiterate	243 (37.7)	351 (62.3)		179 (57.3)	137 (42.7)	
	Primary	277 (45.6)	352 (54.4)		336 (65.4)	196 (34.6)	
	Secondary	898 (66.8)	481 (33.2)		617 (64.6)	359 (35.4)	
	Higher	603 (72.2)	236 (27.8)		548 (60.5)	357 (39.5)	
Residence				0.033			0.018
	Rural	983 (56.3)	633 (43.7)		807 (65.1)	420 (34.9)	
	Urban	1038 (60.7)	787 (39.3)		873 (59.7)	629 (40.3)	
Region				0.762			0.887
	Central	543 (58.4)	360 (41.6)		450 (62.7)	256 (37.3)	
	Southern	480 (60.8)	322 (39.2)		399 (61.0)	274 (39.0)	
	Eastern	571 (58.2)	414 (41.8)		456 (63.6)	269 (36.4)	
	Northern	427 (58.2)	324 (41.8)		375 (62.7)	250 (37.3)	
Marital status				<0.001			<0.001
	Unmarried	224 (83.9)	49 (16.1)		428 (83.9)	90 (16.1)	
	Married	1617 (60.4)	1059 (39.6)		1227 (58.3)	922 (41.7)	
	Widowed/Divorced/Separated	180 (37.8)	312 (62.2)		25 (42.7)	37 (57.3)	
BMI				<0.001			<0.001
	Underweight	213 (72.6)	73 (27.4)		244 (76.6)	74 (23.4)	
	Normal	1048 (66.3)	520 (33.7)		1057 (66.2)	551 (33.8)	
	Overweight	598 (52.6)	571 (47.4)		348 (51.4)	363 (48.6)	
	Obesity	162 (38.6)	256 (61.4)		31 (34.3)	61 (65.7)	

Abbreviation: BMI, body mass index.

The results of the binary logistic regression model reported in Table 3 presented a significant sex‐specific difference in the associated factors of NCDs between men and women. Age, place of residence, and BMI of the respondents were found to be statistically significant associated factors of NCDs among both sexes in Bangladesh. Educational level was also a statistically significant associated factor with NCDs among men and women, although the direction and magnitude of the associations differed by sex.

**TABLE 3:** Sex‐specific effects of the sociodemographic characteristics on noncommunicable diseases (NCDs) status among wealthier households in Bangladesh.

		Women		Men	
Variables	Categories	aOR (95% CI)	*p* value	aOR (95% CI)	*p* value
Age (years)					
	≤18	1		1	
	19–29	1.03 (0.47, 2.25)	0.937	1.47 (0.56, 3.85)	0.437
	30–44	3.18 (1.44, 7.00)	0.004	3.21 (1.21, 8.53)	0.020
	45–59	7.30 (3.15, 16.88)	<0.001	7.47 (2.82, 19.79)	<0.001
	60+	10.20 (4.11, 25.31)	<0.001	12.76 (4.78, 34.08)	<0.001
Education					
	Illiterate	1		1	
	Primary	1.05 (0.80, 1.38)	0.710	1.09 (0.77, 1.55)	0.621
	Secondary	0.73 (0.54, 0.97)	0.029	1.37 (0.97, 1.93)	0.072
	Higher	0.69 (0.50, 0.96)	0.027	1.62 (1.17, 2.25)	0.004
Residence					
	Rural	1		1	
	Urban	1.29 (1.06, 1.58)	0.012	1.32 (1.08, 1.61)	0.007
Region					
	Central	1		1	
	Southern	0.86 (0.67, 1.11)	0.254	0.97 (0.73, 1.28)	0.805
	Eastern	1.08 (0.84, 1.40)	0.549	1.03 (0.79, 1.35)	0.807
	Northern	0.92 (0.70, 1.20)	0.540	0.92 (0.70, 1.20)	0.534
Marital status					
	Unmarried	1		1	
	Married	0.95 (0.64, 1.42)	0.806	1.14 (0.78, 1.67)	0.486
	Widowed/Divorced/Separated	0.99 (0.61, 1.62)	0.996	1.75 (0.88, 3.48)	0.109
BMI					
	Normal	1		1	
	Underweight	0.60 (0.41, 0.88)	0.008	0.67 (0.45, 0.99)	0.046
	Overweight	1.73 (1.39, 2.15)	<0.001	1.80 (1.44, 2.24)	<0.001
	Obesity	3.00 (2.20, 4.10)	<0.001	4.08 (2.19, 7.58)	<0.001

Abbreviation: BMI, body mass index.

It could be noticed from Table 3 that age was significantly associated with NCDs among both men and women. Men aged 30–44 years had a 3.21 times (*p* value = 0.020) increased association of NCDs with respect to men in the age group ≤18. Similarly, women of the 30–44 age group had 3.18 times (*p* value = 0.004) greater association of NCDs compared to women aged ≤18 years. In addition to this, men in the age group of 45–59 and 60+ years had 7.47 times (*p* value < 0.001) and 12.76 times (*p* value < 0.001) higher chance of NCDs compared to men aged ≤18 years, respectively. Again, women (aOR = 1.29 and *p* value = 0.012) and men (aOR = 1.31 and *p* value = 0.007) living in the urban areas were at a greater association of NCDs compared to their counterparts. Although the eastern region had found an increased association of NCDs, this factor was not found to be statistically significant for NCDs.

Overweight and obesity were found to have a statistically significant association with NCDs among both men and women. Overweight men were found to be 1.80 times (*p* value < 0.001) more likely to have NCDs compared to men having a normal BMI, whereas women had a 1.73 times (*p* value < 0.001) increased chance. Similarly, obese men had 4.08 times (*p* value < 0.001) greater likelihood of NCDs compared to men with normal BMI, whereas women had approximately three times (aOR = 3.00 and *p* value < 0.001) higher chance. Men who completed their higher level of education had 62% (aOR = 1.62 and *p* value = 0.004) higher chance of NCDs compared to men with no formal education. In contrast, women with secondary and higher levels of education were 27% (aOR = 0.73, *p* value = 0.029) and 31% (aOR = 0.69, *p* value = 0.027) less likely to experience NCDs compared to women who had no formal education, respectively.

## 4 Discussion

This study aimed to examine the sex‐specific determinants of NCDs among respondents from wealthier households in Bangladesh using data from BDHS 2022. The findings showed that the prevalence of NCDs was higher among women than among men. Age, place of residence, and body mass index were the main factors associated with NCDs in both sexes. These results highlight important sex‐based disparities in the burden and suggest the need for sex‐sensitive public health interventions in Bangladesh.

Age was a key determinant of NCDs in both men and women in our study. Elder people had a higher likelihood of developing NCDs compared to their younger counterparts. Our findings align with a study conducted in Nepal, where the prevalence of conditions, such as hypertension, hyperlipidemia, diabetes, and cardiovascular diseases, increases substantially with advancing age [17]. Aging is associated with physiological changes, including altered lipid metabolism, reduced vascular elasticity, oxidative stress, telomere shortening, and chronic inflammation, all of which contribute to cumulative cellular damage and impaired organ function. These mechanisms help explain why the burden of NCDs rises with age in both sexes [17, 18]. Again, it was also seen that the odds of NCDs were higher among adult women compared to men. A study conducted in India shows that NCDs and multimorbidity are more common in adult women than in men, especially in those 45 years of age and older [19]. The protective effects of estrogen on cardiovascular health are diminished by hormonal changes that occur after menopause, raising the likelihood of heart disease, diabetes, and hypertension. Due to the differing effects of aging on metabolic and inflammatory pathways, women also have higher rates of age‐related NCD escalation [19].

Our study also underscored that both women and men living in urban areas have a higher likelihood of experiencing NCDs compared to those living in rural areas. This finding aligns with several studies conducted in Uganda, Africa, and Bangladesh that, due to the factors associated with urbanization, NCDs, such as hypertension, diabetes, obesity, and metabolic syndrome, are consistently more common in urban residents of both sexes than in rural ones [20, 21, 22].

Moreover, marital status showed an association with NCDs in the unadjusted model; however, this association was not statistically significant after adjustment for other sociodemographic characteristics in both logistic regression models for men and women in this study. Several studies conducted in Somaliland and Bangladesh showed that marital status is associated with higher NCD risks, particularly for widowed, divorced, or separated individuals compared to married or unmarried ones [15, 23]. One of the reasons behind this finding can be that married people benefit from spousal support, partners’ encouragement of better habits, and shared resources that reduce stress and lower the incidence of NCDs, including diabetes and hypertension. Individuals who are single or have been married experience isolation, which results in decreased nutrition, inactivity, and delayed care [24]. Widowed or divorced adults experience chronic stress, depression, and grief, triggering inflammation, cortisol elevation, and metabolic disruptions that promote NCDs. They often adopt riskier habits like smoking or alcohol use without accountability [25].

Disparities in BMI are also evident in our study. We found that men and women who were underweight were less likely to experience NCDs compared to those who had a normal weight. Similarly, overweight or obese people were more likely to experience NCDs. A study from several LMICs and another study from Ethiopia have found that the likelihood of hypertension, diabetes, and NCDs multimorbidity is around two to three times greater in overweight/obese people than in normal‐weight adults. On the other hand, underweight groups in these datasets often have a lower reported prevalence of these cardiometabolic NCDs, even though they may be more associated with other outcomes like mortality, pulmonary disease, or fractures [26, 27, 28]. Insulin resistance, dyslipidemia, hypertension, and systemic inflammation are all directly linked to Type 2 diabetes, cardiovascular disease, some types of cancer, and complicated multimorbidity. Severe obesity is linked to very high risks of obesity‐related multimorbidity, making long‐term obesity from early adulthood especially detrimental [28, 29]. On the other hand, underweight adults have less adipose tissue and lower metabolic load, so the classic obesity‐related NCDs (Type 2 diabetes, hypertension, and hyperlipidemia) are often less common in this group than in normal‐weight or overweight groups [27].

As NCDs are associated with a higher level of functional limitations for both men and women, SDG Target 3.4 has been made to reduce premature mortality from major NCDs by one‑third by 2030, which is supported by WHO's Global Action Plan for NCDs and a monitoring framework with nine global targets [30, 31]. The updated 25 “quick buys” and WHO's “best buys” list cost‐effective measures such as increased taxes on alcohol and tobacco, lowering salt and trans fats, limiting marketing, and developing population‐level CVD/diabetes management plans that prioritize equity and quicker results [32]. In accordance with national Five‐Year Plans and WHO guidelines, Bangladesh has approved a Multisectoral Action Plan for the Prevention and Control of NCDs 2018–2025, which aims to reduce associated factors with NCDs and improve primary care services for cancer, diabetes, chronic respiratory disease, and CVD [33]. The plan also includes tobacco control, promotion of a healthy diet and physical activity, integration of NCD screening and treatment into primary healthcare, and a national NCD communication strategy to reduce high‐risk behaviors [33]. Now it is time for the individual to avoid tobacco and secondhand smoke, maintaining a healthy weight through regular physical activity, limiting harmful alcohol use, improving diet quality (higher intake of fruits, vegetables, and whole grains with reduced salt, sugar, trans‐fat, and ultra‐processed foods), and routinely monitoring blood pressure, blood glucose, and lipid levels, along with adherence to recommended treatments and seeking medical attention early. In terms of how NCDs affect the healthcare system, the reviewed studies indicate that NCD‐related healthcare costs are continuously rising, further taxing public finance [34]. In LMICs, where funding does not keep up with the demand for NCD‐related care, this financial strain is particularly noticeable [35]. Men and women experience these effects differently, and the health system must act to address them. To address the health needs of men and women with sex‐sensitive guidelines and protocols and support their implementation, these actions include enhancing the ability of healthcare providers to recognize and address sex‐related issues in the provision of health services and programs [36]. To develop strategies aimed at reducing sex‐associated disparities in access to and use of health services, it is also necessary to identify specific barriers to the use of NCD prevention and treatment services by men and women, including cultural norms that influence healthcare‐seeking and treatment behaviors among men, women, boys, and girls in the community. These strategies should address the particular disadvantages that women and girls typically face that make them less likely to access NCD services in certain communities, such as their lack of decision‐making authority and their financial incapacity to access public health policy efforts in the prevention and treatment of NCDs [36].

### 4.1 Strengths and Limitations

This study has some notable strengths, such as a sex‐specific analysis of NCDs, which provided a clear comparison of determinants of NCDs between men and women. In addition, the use of appropriate survey design considerations strengthened the robustness of the study. However, this study has some limitations that should be mentioned. First, the causal relationship between sociodemographic factors and NCDs cannot be established as the analysis was based on cross‐sectional data. Second, the study focused only on the respondents from wealthier households, which restricts the generalizability of the findings to the entire Bangladeshi population. Future research should include all wealth quintiles to enable comparative analyses and provide a more comprehensive understanding of socioeconomic inequalities in NCDs. Third, important behavioral variables, such as smoking status, exercise status, and drinking habit (tea or coffee), were not included in this study due to insufficient data. Fourth, potentially important behavioral and metabolic factors, such as tobacco use, alcohol consumption, physical activity, and dietary factors, were not available in the BDHS 2022 dataset used in this study and, therefore, could not be included in the regression models. The omission of these factors may have resulted in residual or unmeasured confounding and may partly influence the observed sex‐specific associations. Therefore, the reported associations should not be interpreted as causal effects. Future studies incorporating a broader range of behavioral, metabolic, and dietary factors are warranted to better understand the determinants of sex‐specific differences in NCDs. Finally, the definition of NCDs in this study was limited to hypertension and diabetes due to data availability in the BDHS 2022. Information on other major NCDs (such as cardiovascular diseases, chronic respiratory diseases, and cancers) was not collected in the survey and, therefore, could not be included in the analysis. Future research should include all possible conditions to construct a comprehensive national estimate of the burden of NCDs.

## 5 Conclusion

This study revealed a high burden of NCDs among the wealthier individuals in Bangladesh, with a greater prevalence among women compared to men. Some key sociodemographic factors, such as age, education, place of residence, and BMI, were found to have statistically significant associations with NCDs for both men and women. Adult age (30–44, 45–59, and 60+ years), urban place of residence, and higher BMI (overweight and obese) were associated more with the development of NCDs among men than women. Moreover, highly educated men had a greater association with NCDs. In contrast, secondary and higher levels of education played a role in less likely to have of NCDs among women. These findings highlight the importance of considering sex‐specific patterns when designing strategies for NCD prevention and control among wealthier populations in Bangladesh.

## Author Contributions

Md. Ismail Hossain generated the idea of this study and shared it with Sourav Roy. Sourav Roy performed the statistical analysis. Sourav Roy and Raisha Binte Islam drafted the manuscript. Sourav Roy, Raisha Binte Islam, and Syed Toukir Ahmed Noor contributed to reviewing and editing the manuscript with Md. Ismail Hossain. Md. Ismail Hossain supervised the entire work. All authors read and approved the final manuscript.

## Funding

The authors have nothing to report.

## Conflicts of Interest

The authors declare no conflicts of interest.

## Data Availability

The dataset used in this study is publicly available from the Bangladesh Demographic and Health Survey (BDHS) 2022, upon reasonable request through the DHS program (https://www.dhsprogram.com/data/available‐datasets.cfm/). However, the final dataset used in this study will be available upon request to the authors.
